# Gut microbiota and their relationship with circulating adipokines in an acute hepatic encephalopathy mouse model induced by surgical bile duct ligation

**DOI:** 10.1016/j.heliyon.2024.e38534

**Published:** 2024-09-26

**Authors:** Bokyung Lee, Danbi Jo, Jihyun Park, Oh Yoen Kim, Juhyun Song

**Affiliations:** a Department of Food Science and Nutrition, Dong A University, Sahagu, Nakdongdaero 550 beon-gil, 49315, Busan, Republic of Korea; b Department of Anatomy, Chonnam National University Medical School, Hwasun, 58128, Jeollanam-do, Republic of Korea; c Department of Health Sciences, Graduate School of Dong-A University, Sahagu, Nakdongdaero 550 beon-gil, 49315, Busan, Republic of Korea

**Keywords:** Bile duct ligation, Gut microbiota, Adipo-myokine, Hepatic encephalopathy

## Abstract

**Background and aims:**

Various studies have shown the importance of the gut microbiota in human health. However, little is known about gut microbiome patterns and their effect on circulating adipo-myokine levels in hepatic encephalopathy (HE). We investigated the relationship between the gut microbiota and adipo-myokine levels using a mouse model of HE induced by surgical bile duct ligation (BDL).

**Methods and results:**

Wild-type C57BL/6J mice were subjected to sham surgery or BDL. Severe body weight loss, suppressed feed intake, and liver failure were observed in BDL mice compared with sham control mice. Additionally, changes in gut microbial communities and serum adipo-myokine levels were noted in BDL mice. In the BDL mouse gut, we identified 15 differentially abundant taxa including the phylum *Verrucomicrobiota*, the classes *Actinomycetes* and *Verrucomicrobiae*, the order *Verrucomicrobiales*, the families *Akkermansiaceae, Bacteroidaceae*, *Rikenellaceae*, and *Oscillospiraceae*, the genera *Alistipes, Akkermansia*, *Muribaculum*, and *Phocaeicola*, and the species *Akkermansia muciniphila*, *Alistipes okayasuensis*, and *Muribaculum gordoncarteri* by LEfSe analysis (LDA score≥4.0). Higher levels of certain adipo-myokines such as BDNF were detected in the serum of BDL mice. Spearman correlation analysis revealed that certain adipo-myokines (e.g., FSTL1) were positively correlated with the class *Actinomycetes*, the family *Rikenellaceae*, the genus *Alistipes*, and the species *Alistipes okayasuensis*. Interestingly, *A. okayasuensis* and *M. gordoncarteri*, recently isolated microbes, showed richness in the gut of BDL mice and demonstrated positive correlations with adipo-myokines such as FGF21.

**Conclusions:**

Overall, our results suggest that alteration of the gut microbiota in patients with HE may be closely correlated to the levels of adipo-myokines in the blood.

## Highlights

• BDL mice show higher proportions of mucus-colonizing bacteria such as *A. muciniphila* in gut.
• BDL surgery leads to enrichment of *Oscillospiraceae*, *Akkermansiaceae*, *Bacteroidaceae*, and *Rikenellaceae* in gut.
• *A. muciniphila* is positively correlated with LIF involved in metabolic regulation, leading to weight loss.
• Adipo-myokines such as BDNF, FGF21, and LIF are significantly higher in BDL mice blood.
• Eight taxa show positive correlations with more than 5 factors among the detected adipo-myokines.

## Graphical abstract

**Figure:**
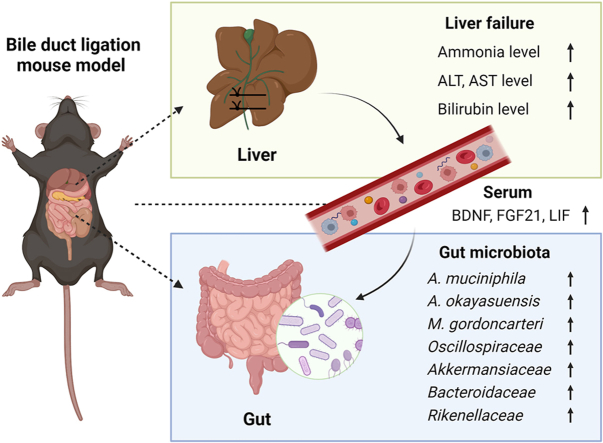

## 1 Introduction

Hepatic encephalopathy (HE) brought on by severe liver diseases such as liver cirrhosis is mainly characterized by neurological dysfunction, including cognitive dysfunction, anxiety, depression, and coma [1]. Systemic metabolic dysfunction, high levels of ammonia, and neuroinflammation are the main causes of HE [2,3]. In addition, it has been reported that more than 50 % of patients with hepatic cirrhosis can develop sarcopenia, indicating the loss of muscle mass and function [4]. Sarcopenia is a major complication in severe liver diseases such as cirrhosis due to altered glucose metabolism, lipid oxidation, ketogenesis, and increased protein catabolism, leading to the loss of adipose and muscle tissue [4].

Previous studies have reported the relationship between the gut microbiota and liver failure, suggesting that manipulating the gut microbiota is an emerging approach for the treatment of liver diseases [5,6]. The gut microbiota plays a critical role in immune responses and homeostasis of physiological functions in the body [7]. In the gut, the intestinal barrier, composed of biofilms and epithelial cells, is characterized by immunological protection and absorptive functions [8]. Impaired intestinal barrier and gut homeostasis allow bacteria and toxic substances into the systemic blood circulatory system [9]. In the dysbiosis of microbiota, the intestinal barrier is disrupted with increased blood vessel permeability under metabolic imbalance conditions such as obesity and metabolic dysfunction-associated fatty liver disease (MAFLD) [10]. Bile acids are known to regulate the microbiota composition, and primary bile acids are converted to secondary bile acids through the microbiome in the gut [11]. A recent study has shown that bile acid modification could reduce the production of gut peptides and short-chain fatty acids accompanied by microbiome dysbiosis [12].

Since Liu et al. first reported the relationship between the gut microbiota pattern and pathological progress of HE [13], several clinical studies have suggested the different distribution of the intestinal microbiota (i.e., *Alcaligenaceae*, *Lachnospiraceae*, and *Porphyromonadaceae*) between patients with HE and normal subjects, which are involved in severe pathological processes [14]. Although there is considerable evidence on the alteration of the gut microbiota during liver failure, there is no report identifying a specific gut microbe and its relevance to blood metabolic parameters in HE. In addition, the gut microbiota modifies the profiles of adipokines and myokines by regulating their secretion, leading to metabolic imbalance [15]. G protein-coupled bile acid receptor 1, a bile acid membrane receptor on intestinal L-cells, could be activated by the secretion of glucagon-like peptide 1, which was reported to improve liver function and glucose metabolism in obese mice [16]. In addition, farnesoid X receptor, a nuclear receptor, acts as the main regulator of insulin sensitivity through enteroendocrine regulation [17]. A study indicated that primary bile acid supplementation could affect the gut microbiota composition and the level of adiponectin, a type of adipokine [18]. Other studies reported that alteration of the gut microbiota could promote lower fat mass and higher muscle mass accompanied by changes in the levels of leptin [19,20] and adiponectin [21]. Although there is considerable evidence on the relationship between adipo-myokine secretion and bile acids/liver function, their association has not been fully explored.

Here, we examined the gut microbiota composition and their relevance to blood metabolic biomarkers, particularly adipo-myokine levels, in a mouse model of HE commonly induced by surgical bile duct ligation (BDL) [22].

## 2 Materials and methods

### 2.1 Animal model

Wild-type C57BL/6J mice (n = 14, 12-week-old male mice; Koatech, Republic of Korea) were housed following the guidelines of the Laboratory Animal Research Center of Chonnam National University (CNU) under a 16 h light and 8 h dark cycle at approximately 23 °C with 60 ± 10 % humidity and given ad libitum access to water and normal chow.

### 2.2 Experimental design

The mice were anesthetized with 2,2,2-tribromoethanol/2-methyl-2-butanol (Sigma-Aldrich) at 0.2 mg/g body weight. Then, the mice were randomly subjected to sham surgery (sham control mice, n = 7) or BDL (BDL mice, n = 7) to induce HE. BDL was performed under 1.5–2% isoflurane anesthesia (in a mixture of oxygen), and the bile duct was ligated using a 5-0 black silk suture. On the 14th postoperative day (2 weeks after surgery), the mice were sacrificed for the sampling of blood after the collection of feces. Body weight and food intake were measured every day for 2 weeks after BDL surgery until the time point of sacrifice. All experiments were performed following the “96 Guidance for Animal Experiments” recommendation approved by the Animal Ethics Committee at CNU. The protocols were approved by the Animal Ethics Committee at CNU (CNU IACUC-H-2022-8).

### 2.3 Ammonia detection assay

Ammonia levels in the mouse plasma were measured using an ammonia assay kit (ab83360; abcam, Cambridge, UK) according to the manufacturer's instructions. The plasma was mixed with an ammonia reaction mixture (Ammonia assay buffer, OxiRed probe, Developer, Enzyme mix, and Converting enzyme) and incubated for 1 h at 37 °C in the dark. After incubation, ammonia levels were detected at 570 nm using an Epoch microplate reader (BioTeck, Winooski, VT, USA).

### 2.4 Total and direct bilirubin detection assay

Bilirubin (total and direct) levels in the mouse plasma were detected using a bilirubin colorimetric assay kit (K553-100; BioVision, Milpitas, CA, USA) according to the manufacturer's instructions. The plasma was mixed with the Catalyst and Reagent Mix and subsequently incubated for 30 min at room temperature. After incubation, the Total Bilirubin Probe or Direct Bilirubin Probe solution was added to the samples and further incubated for 20 min at room temperature in the dark. After incubation, the total bilirubin level was measured at 600 nm, and the direct bilirubin level was measured at 550 nm using an Epoch microplate reader.

### 2.5 Liver function assay

Plasma levels of alanine aminotransferase (ALT, ab282882; abcam) and aspartate amino aminotransferase (AST, ab263882; abcam) were measured with an ELISA kit according to the manufacturer's instructions. The plasma was mixed with an ALT antibody cocktail or AST antibody cocktail and incubated for 1 h at room temperature. After incubation, TMB development solution was added and incubated for 10 min at room temperature in the dark. After incubation, stop solution was added, and ALT and AST levels were measured at 450 nm using an Epoch microplate reader.

### 2.6 Serum adipo-myokine panel assay

Serum myokine levels were measured using the Mouse Myokine Magnetic Bead Panel assay kit (MILLIPLEX®; Merck Millipore, Billerica, MA, USA). MILLIPLEX assay was performed on the Luminex platform (Luminex, Austin, TX, USA), and the median fluorescence intensity (MFI) was determined. A total of 11 types of myokines were analyzed: brain-derived neurotrophic factor (BDNF), fibroblast growth factor 21 (FGF21), follistatin-like protein 1 (FSTL1), interleukin-15 (IL-15), interleukin-6 (IL-6), irisin, leukemia inhibitory factor (LIF), myostatin (MSTN), oncostatin M (OSM), osteocrin/musclin (OSTN), and secreted protein acidic and rich in cysteine (SPARC; osteonectin; BM40). The analysis was performed in triplicate, and the average concentration was calculated.

### 2.7 Intestinal microbiota analysis

Snap-frozen fecal samples (n = 13: sham control mice, n = 7; BDL mice, n = 6) were sent to Macrogen Inc. (Seoul, Korea) for metagenome sequencing. The sample of BDL mouse #5 was not collected due to an insufficient amount of feces. Genomic DNA was extracted from the samples with the DNeasy PowerSoil Kit (Qiagen, Hilden, Germany) and quantified by Quant-IT PicoGreen (Invitrogen, Waltham, MA, USA). The V3 to V4 regions of the 16S rRNA gene were amplified using the barcoded primers F341 (5′-TCGTCGGCAGCGTCAGATGTGTATAAGAGACAGCCTACGGGNGGCWGCAG-3′) and R805 (5′-GTCTCGTGGGCTCGGAGATGTGTATAAGAGACAGGACTACHVGGGTATCTAATCC-3′). The PCR products were purified and used for DNA library preparation. Paired-end (2 × 300 bp) sequencing was performed using the MiSeq™ platform (Illumina, San Diego, CA, USA) at Macrogen Inc. (Seoul, Korea). Next, 16S rRNA gene sequence analysis was performed with QIIME 2 version 2023.2 [23]. Demultiplexed paired-end FASTQ files were imported, and feature table construction and chimera removal were performed for each run using the DADA2 method and the denoise-single option with default settings; however, the first 250 bases and low-quality bases after position 200 were truncated [24]. Feature tables and sequences were merged with the feature-table merge and feature-table merge-seqs plugins, followed by de novo read alignment with MAFFT [25]. Unconserved and gapped alignments were filtered by the alignment mask plugin with default parameters. A phylogenetic tree was created with FastTree MP (version2.1.10) using the filtered alignment method. Principal coordinate analysis (PCoA) was performed using q2‐diversity after the samples were rarefied (subsampled without replacement) to 5000 sequences per sample. For taxonomic assignment, a custom classifier was trained based on the truncated sequence reads (231 bases) against the Greengenes database (version 13.8). The sequences are available in the NCBI BioProject database with the project identification number PRJNA983098 (sample accession numbers: SAMN36269388 to SAMN36269399).

### 2.8 Statistical analysis

Statistical analysis was performed using SPSS 27.0 (IBM Corp., Armonk, NY, USA). The results of *t*-test are expressed as the mean ± standard error (SE). The significance of the difference was evaluated by Mann-Whitney *U* test. Linear discriminant analysis effect size (LEfSe) analysis was conducted to identify specific taxa for each treatment group. In addition, significantly different taxa between the groups were identified using both Mann-Whitney *U* test and LEfSe analysis. Multiple alignment between amplicon sequence variants (ASVs) was generated with MAFFT (v7.475) [25], and a phylogenetic tree was generated with FastTree (v2.1.10) [26]. Graphical outputs of the 16sRNA gene and correlation map of adipo-myokines were generated with the ggplot (v3.2.1) package in R program. Linear discriminant analysis (LDA) between the samples and control at the phylum, family, and species levels was performed, and differences were evaluated by Mann-Whitney *U* test with adjustment for multiple testing according to the method of Benjamini and Hochberg [27]. Correlation between the gut microbiota and adipo-myokines was examined by Spearman correlation analysis, and a hierarchical clustering heatmap was generated using XLSTAT. Spearman correlation analysis was conducted to assess the relationship between the variables. A *p* value of less than 0.05 was considered statistically significant.

## 3 Results

### 3.1 Effect of surgical BDL on body weight loss and feed intake changes

**Fig. 1:**
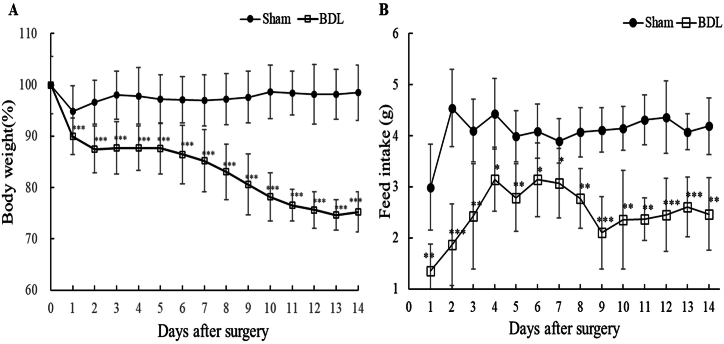
Changes in body weight (A) and feed intake (B) between sham control and BDL mice. Data are presented as the mean ± SD. ∗*p* < 0.05, ∗∗*p* < 0.01, ∗∗∗*p* < 0.001 vs. sham control; tested by two-tailed Student's t-test. Surgery was conducted on day 0, and mice were sacrificed on day 14. The body weight and food intake of all mice were assessed daily. Sham: control mice that received sham surgery (n = 7); BDL: mice that received BDL surgery (n = 6).

We monitored the body weight and feed intake of wild-type C57BL/6J mice that received sham surgery (shame control mice) or BDL (BDL mice) daily until they were sacrificed (Fig. 1). The body weight of BDL mice was significantly reduced, whereas the body weight of sham control mice remained constant until they were sacrificed (Fig. 1A). Most BDL mice lost around 29.2 % of their total body weight within 14 days after surgery except for 2 mice (BDL mouse #3 and #4), which showed a weight loss of around 14.7 % (Supplementary Fig. 1).

Fig. 1B shows the feed intake (g) of both sham control and BDL mice for 2 weeks. The average food intake rate was higher in the sham control group than in the BDL group for 2 weeks after surgery (Fig. 1B). Overall, significantly lower feed intake was observed among BDL mice compared with sham control mice. After surgery, sham control mice consumed feed at an average rate of 4.1 ± 0.56 g/day. In comparison with sham control mice, BDL mice showed 39 % less feed intake with some fluctuations.

### 3.2 Assessment of the liver function parameters of BDL mice

**Fig. 2:**
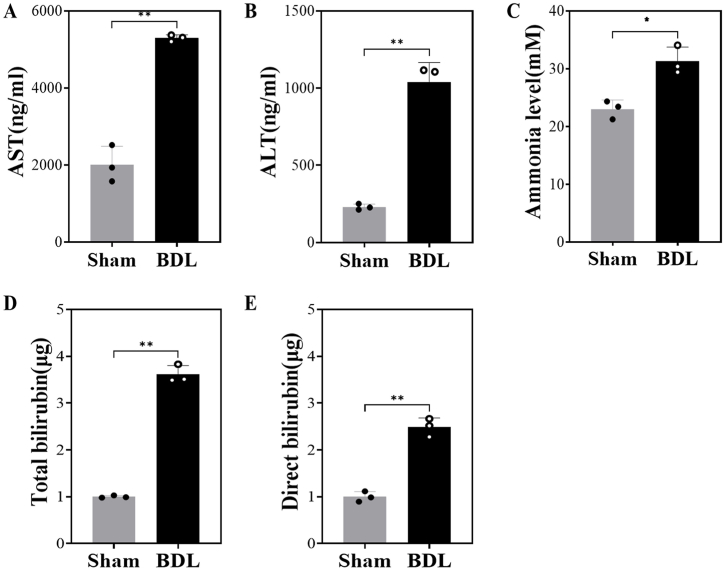
Liver function and HE-related biomarkers of sham control and BDL mice. Data are presented as the mean ± SEM. ∗*p* < 0.05, ∗∗*p* < 0.01 vs. sham control; tested by two-tailed Student's t-test. Liver function and HE biomarkers such as serum levels of AST (A), ALT (B), ammonia (C), total bilirubin (D), and direct bilirubin (E) were significantly higher in BDL mice than in sham control mice on the 14th day after BDL. Sham: control mice that received sham surgery (n = 3); BDL: mice that received BDL surgery (n = 3).

To examine the development of liver failure, the biochemical parameters of liver function were measured at 14 days after surgery (Fig. 2). Due to the shortage of plasma samples, the parameters were measured in available samples of each group (sham control mice, n = 3; BDL mice, n = 3). As shown in Fig. 2, the average values of liver function parameters were significantly different between the two groups, and furthermore, the parameter levels within the same group were shown constant. Overall, plasma levels of ALT and AST, as well as total and direct bilirubin, were markedly elevated in BDL mice compared with sham control mice (Fig. 2A, B, 2D, and 2E). In addition, ammonia levels were increased in BDL mice (31.3 ± 2.0 mM) compared with sham control mice (23.0 ± 1.3 mM, *p* < 0.01) (Fig. 2C). The average level of ALT was 4.5-fold higher in BDL mice (1037.13 ± 128.14 ng/mL) than in sham control mice (230.13 ± 196.22 ng/mL, *p* = 0.0004) (Fig. 2A). The activity of plasma AST was also significantly increased by 2.6-fold in BDL mice compared with sham control mice (Fig. 2B). The average levels of total and direct bilirubin were increased by 3.61-fold and 2.49-fold, respectively, in the plasma of BDL mice compared with sham control mice (Fig. 2D and E). These results indicated that liver dysfunction was successfully induced by surgical BDL in the mice.

### 3.3 Alteration of the gut microbiota composition in BDL mice

**Fig. 3:**
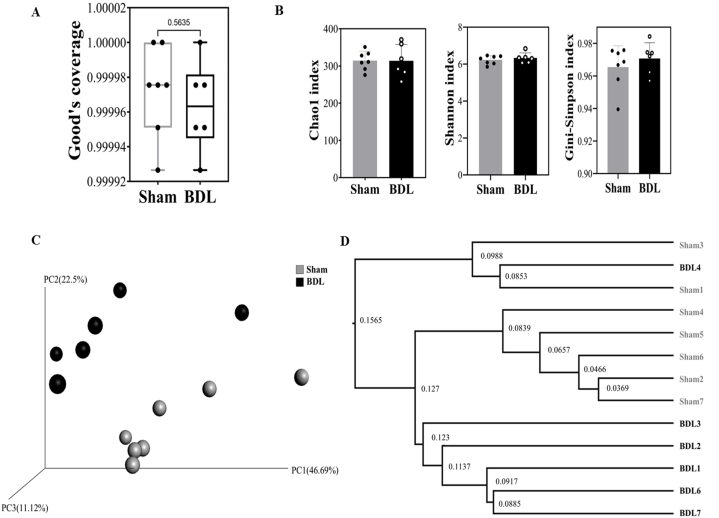
Effect of BDL on the community structure and diversity of the mouse gut microbiota. The box plots show differences in the fecal microbiota diversity indices between sham control and BDL mice, which were assessed by the (A) Good's coverage diversity index, as well as the (B) Shannon, Chao1, and Gini-Simpson indices. Tested by Mann-Whitney *U* test. The level of similarity between the fecal microbial communities detected in the BDL (black) and sham (gray) groups was assessed by (C) principal coordinate analysis (PCoA; based on the weighted UniFrac distance matrix) and the (D) UPGMA tree. Each box plot represents the median, interquartile range, and minimum and maximum values, and the bar plot represents the mean ± SD. Sham: control mice that received sham surgery (n = 7); BDL: mice that received BDL surgery (n = 6).

Gut microbiota perturbation by surgical BDL was observed at the phylum, family, and species levels. To investigate the effect of surgical BDL on the richness, diversity, and composition of the gut microbiota in mice, fresh fecal samples were collected from the mice on the day of sacrifice, and their bacterial diversity was assessed by 16S rRNA sequencing. From 13 fecal samples (sham control mice, n = 7; BDL mice, n = 6), a total of 1,173,606 high-quality reads were clustered in 995 ASVs at 85 % identity after removing unqualified sequences. The sequencing coverage indices of the two groups were greater than 0.99 (Fig. 3A), indicating that the number of sequencing reads in the groups was statistically sufficient. The alpha-diversity of the fecal microbiota collected from the mice was evaluated and showed that there was no significant difference between BDL and sham control mice (Fig. 3B, Supplementary Fig. 2). However, bacterial beta-diversity differed according to treatment as demonstrated by PCoA of the weighted Fast UniFrac distance matrix and the UPGMA tree (Fig. 3C and D). Notably, 2 BDL mice with the lowest weight loss were clustered more closely to sham control mice in beta-diversity analysis (Fig. 3C and D, Supplementary Fig. 1).

### 3.4 Specific bacteria enriched in the gut of BDL mice

**Fig. 4:**
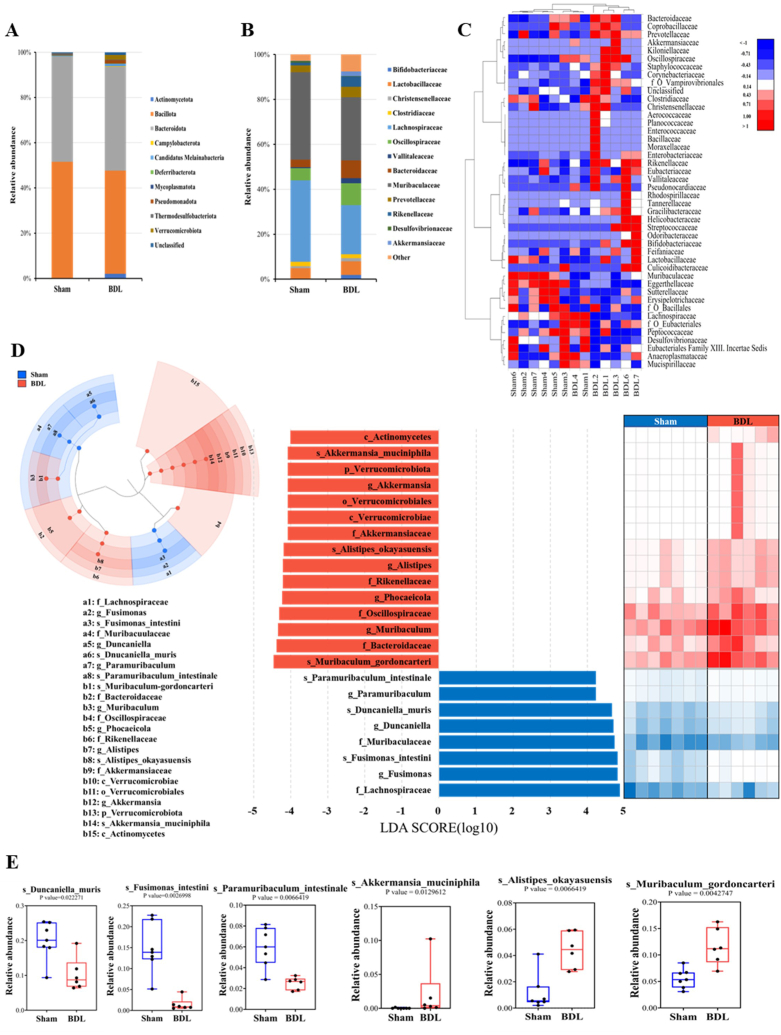
Effect of BDL on the composition of the mouse gut microbiota. The average relative abundances of each treatment group at the phylum (A), and family (B; Top 10) levels were determined. The color gradient indicates the proportion of the bacteria, and the left side of the figure shows the names of taxa represented by the color gradient. (C) Heatmap showing the relative abundance of the gut microbiota at the family level for each mouse. The increase in the relative abundance of the gut microbiota is represented by a transition from blue to white to red, as shown in the legend. (D) Cladogram of linear discriminant analysis (LDA) effect size (LEfSe) analysis of microbial abundance from the phylum to species level. The LDA score represents the log changes in relative bacterial family representation in each group. The LDA score was set to ≥4.0. (E) Relative abundance of the gut microbiota at the species level compared by LDA score ≥4.0. Sham: control mice that received sham surgery (n = 7); BDL: mice that received BDL surgery (n = 6).

We identified perturbed gut microbiota at the phylum, family, and species levels in BDL mice and further validated the results by LEfSe analysis (Fig. 4D). A total of 11 phyla were detected in all the samples examined in this study (Fig. 4A–Table 1). The most abundant taxa identified in the fecal samples were *Bacillota* (Firmicutes) and *Bacteroidota* (Bacteroidetes), accounting for 98 % and 91 % of the total sequences from sham control and BDL mice, respectively. Specifically, a reduced Firmicutes/Bacteroidetes (F/B) ratio and increased taxonomic diversity were observed in BDL mice at the phylum level (Fig. 4A).

**Table 1:** Significant bacterial taxa of the gut microbiota at the species level in sham control and BDL mice.

Group	Phylum	Class	Order	Family	Genus	Species	Relative median % abundance		*p* value
							Sham	BDL	
Sham	Actinomycetota	Coriobacteriia	Eggerthellales	Eggerthellaceae	Adlercreutzia	Adlercreutzia caecimuris	0.05	0.00	0.01
	Bacillota	Clostridia	Eubacteriales	Clostridiaceae	Butyricicoccus	Butyricicoccus pullicaecorum	0.21	0.04	0.00
				Eubacteriaceae	Eubacterium	Eubacterium oxidoreducens	0.02	0.00	0.03
				Eubacteriales Family XIII. Incertae Sedis	Sinanaerobacter	Sinanaerobacter chloroacetimidivorans	0.03	0.00	0.00
					Zhenpiania	Zhenpiania hominis	0.03	0.01	0.01
				Lachnospiraceae	Anaerotignum	Anaerotignum aminivorans	0.11	0.02	0.04
					Anaerotignum	Anaerotignum lactatifermentans	0.45	0.04	0.00
					Fusimonas	Fusimonas intestini	13.88	0.86	0.00
					Jingyaoa	Jingyaoa shaoxingensis	0.71	0.02	0.03
					Lachnoclostridium	Lachnoclostridium pacaense	0.09	0.00	0.00
					Lachnoclostridium	Lachnoclostridium urinimassiliense	0.04	0.00	0.02
					Lachnoclostridium	[Clostridium] scindens	0.36	0.06	0.00
					Roseburia	Roseburia intestinalis	0.20	0.00	0.05
					Roseburia	Roseburia porci	0.39	0.02	0.01
					Sporofaciens	Sporofaciens musculi	1.11	0.24	0.00
					Variimorphobacter	Variimorphobacter saccharofermentans	0.07	0.02	0.04
				Oscillospiraceae		[Clostridium] methylpentosum	0.11	0.06	0.03
						[Clostridium] viride	0.24	0.09	0.00
					Anaerotruncus	Anaerotruncus colihominis	0.12	0.03	0.00
					Scatolibacter	Scatolibacter rhodanostii	0.02	0.00	0.03
				Peptococcaceae	Peptococcus	Peptococcus simiae	0.03	0.00	0.00
				Vallitaleaceae	Petrocella	Petrocella atlantisensis	0.01	0.00	0.02
		Erysipelotrichia	Erysipelotrichales	Erysipelotrichaceae	Longibaculum	Longibaculum muris	0.03	0.00	0.05
					Tannockella	Tannockella kyphosi	0.01	0.00	0.02
	Bacteroidota	Bacteroidia	Bacteroidales	Muribaculaceae	Duncaniella	Duncaniella muris	20.09	8.72	0.01
					Muribaculum	Muribaculum intestinale	1.45	0.32	0.00
					Paramuribaculum	Paramuribaculum intestinale	5.99	2.68	0.00
					Sangeribacter	Sangeribacter muris	2.31	1.10	0.00
	Pseudomonadota	Betaproteobacteria	Burkholderiales	Sutterellaceae	Parasutterella	Parasutterella excrementihominis	0.17	0.02	0.03
	Thermodesulfobacteriota	Desulfovibrionia	Desulfovibrionales	Desulfovibrionaceae	Desulfovibrio	Desulfovibrio porci	0.57	0.07	0.00
BDL	Actinomycetota	Actinomycetes	Bifidobacteriales	Bifidobacteriaceae	Bifidobacterium	Bifidobacterium pseudolongum	0.00	1.16	0.01
		Actinomycetes	Mycobacteriales	Corynebacteriaceae	Corynebacterium	Corynebacterium casei	0.00	0.04	0.02
	Bacillota	Bacilli	Lactobacillales	Lactobacillaceae	Ligilactobacillus	Ligilactobacillus apodeme	0.15	0.58	0.02
		Clostridia	Eubacteriales		Evtepia	Evtepia gabavorous	0.00	0.13	0.00
				Christensenellaceae	Luoshenia	Luoshenia tenuis	0.01	0.06	0.02
				Clostridiaceae	Butyricicoccus	Butyricicoccus porcorum	0.01	0.02	0.02
				Feifaniaceae	Feifania	Feifania hominis	0.11	0.21	0.04
				Gracilibacteraceae	Gracilibacter	Gracilibacter thermotolerans	0.03	0.11	0.02
				Lachnospiraceae	Lachnoclostridium	[Clostridium] polysaccharolyticum	0.00	1.87	0.04
				Oscillospiraceae	Congzhengia	Congzhengia minquanensis	0.01	0.33	0.00
					Ruminococcus	Ruminococcus champanellensis	0.11	0.88	0.00
				Vallitaleaceae	Vallitalea	Vallitalea pronyensis	0.15	0.64	0.02
	Bacteroidota	Bacteroidia	Bacteroidales	Bacteroidaceae	Bacteroides	Bacteroides faecichinchillae	0.01	0.09	0.01
					Bacteroides	Bacteroides intestinalis	0.00	0.28	0.02
				Muribaculaceae	Muribaculum	Muribaculum gordoncarteri	5.33	11.20	0.00
				Rikenellaceae	Alistipes	Alistipes okayasuensis	0.55	4.45	0.00
				Tannerellaceae	Parabacteroides	Parabacteroides goldsteinii	0.03	0.16	0.01
	Candidatus Melainabacteria		Vampirovibrionales		Vampirovibrio	Vampirovibrio chlorellavorus	0.37	0.81	0.00
	Pseudomonadota	Gammaproteobacteria	Enterobacterales	Enterobacteriaceae	Escherichia	Escherichia fergusonii	0.00	0.03	0.00
	Verrucomicrobiota	Verrucomicrobiae	Verrucomicrobiales	Akkermansiaceae	Akkermansia	Akkermansia muciniphila	0.00	0.39	0.01

Tested by Mann-Whitney *U* test.

To identify specific bacterial taxa with changes in the abundance in each group, the 10 most abundant bacterial taxa in each treatment group were annotated at the family level (Fig. 4B). In both BDL and sham control mice, *Muribaculaceae* was the most abundant (BDL 29.9 %; sham 43.2 %), followed by *Lachnospiraceae* (BDL 18.1 %; sham 33.4 %) and *Oscillospiraceae* (BDL 10.2 %; sham 4.8 %) (Fig. 4B). Specifically, the abundances of *Bifidobacteriaceae* (*p* = 0.009), *Oscillospiraceae* (*p* = 0.013), *Vallitaleaceae* (*p* = 0.002), *Rikenellaceae* (*p* = 0.005), and *Akkermansiaceae* (*p* = 0.013) were significantly increased, whereas those of *Lachnospiraceae* (*p* = 0.04) and *Desulfovibrionaceae* (*p* = 0.001) were significantly reduced in the microbiota of BDL mice compared with sham control mice. In addition, the relative abundances of all detected families in this study were visualized by a heatmap to show the fecal microbiota composition of individual mice (Fig. 4C). Overall, the composition of high-abundance families in the gut microbiota was considerably different between sham control and BDL mice, except for BDL mouse #4. Specifically, the fecal microbiota composition of BDL mouse #4 was similar to that of sham control mice due to the enrichment of the family *Lachnospiraceae* and the lack of the family *Rikenellaceae*, which includes the species *Alistipes okayasuensis*.

At the species level, we identified 50 taxa with significant differences in the relative abundance when two treatment groups were compared (Table 1). The most abundant bacterial taxa in the samples of BDL mice were *Muribaculum gordoncarteri* (11.2 %), *Alistipes okayasuensis* (4.45 %), *Clostridium polysaccharolyticum* (1.87 %), and *Bifidobacterium pseudolongum* (1.16 %) (Table 1). In the samples of sham control mice, several taxa were also detected at relatively high abundances, including the species *Duncaniella muris* (20.09 %), *Fusimonas intestini* (13.88 %), *Paramuribaculum intestinale* (5.99 %), and *Sangeribacter muris* (2.31 %) (Table 1).

Moreover, the gut microbiota composition of both groups was compared by LEfSe analysis to identify specific bacterial taxa enriched in each treatment group. Taxa with a LDA score >4.0 were considered as significantly enriched taxa (Fig. 4D and E). At the taxonomic level, the gut microbiota of BDL mice was enriched in the phylum *Verrucomicrobiota*, the classes *Actinomycetes* and *Verrucomicrobiae*, the order *Verrucomicrobiales*, the families *Akkermansiaceae*, *Bacteroidaceae, Rikenellaceae*, and *Oscillospiraceae*, the genera *Muribaculum, Phocaeicola*, *Alistipes*, and *Akkermansia*, and the species *Muribaculum gordoncarteri*, *Alistipes okayasuensis*, and *Akkermansia muciniphila.* However, the microbiota of sham control mice was enriched in the family *Lachnospiraceae*, particularly the genus *Fusimonas*. Furthermore, we observed the enrichment of the family *Muribaculaceae* with the genera *Duncaniella* and *Paramuribaculum*. At the species level, *Duncaniella muris*, *Fusimonas intestini*, and *Paramuribaculum intestinale* were abundant in the gut of sham control mice (Supplementary Fig. 3).

### 3.5 Serum adipo-myokine levels in sham control and BDL mice

**Fig. 5:**
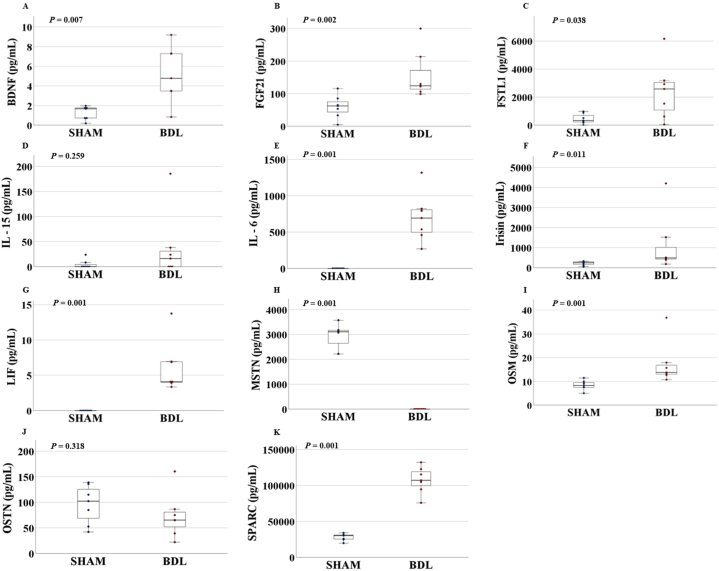
Serum adipo-myokine levels (pg/mL) in sham control and BDL mice. (A) BDNF, (B) FGF21, (C) FSTL1, (D) IL-15, (E) IL-6, (F) Irisin, (G) LIF, (H) MSTN, (I) OSM, (J) OSTN, and (K) SPARC. Data are presented as the mean ± SE; tested by Mann-Whitney *U* test. BDL, bile duct ligation; BDNF, brain-derived neurotrophic factor; FGF21, fibroblast growth factor 21; FSTL1, follistatin-like protein 1; IL-15, interleukin-15; IL-6, interleukin-6; LIF, leukemia inhibitory factor; MSTN, myostatin; OSM, oncostatin M; OSTN, osteocrin/musclin; SPARC, secreted protein acidic and rich in cysteine. Sham: control mice that received sham surgery (n = 7); BDL: mice that received BDL surgery (n = 6).

Fig. 5 shows the serum levels of adipo-myokines in sham control and BDL mice on the 14th day after surgery. A few markers were not detected at all (e.g., IL-6 and LIF in sham control mice; MSTN in BDL mice), which were converted to zero and used for further analysis. Serum levels of BDNF, FGF21, FSTL1, IL-6, irisin, LIF, OSM, and SPARC were significantly higher in BDL mice than in sham control mice (*p* = 0.007, *p* = 0.002, *p* = 0.038, *p* = 0.001, *p* = 0.011, *p* = 0.001, *p* = 0.001, and *p* = 0.001, respectively). On the other hand, the level of MSTN was significantly lower in BDL mice than in sham control mice (*p* = 0.001). However, the levels of IL-15 and OSTN were not significantly different between the two groups (*p* = 0.259 and *p* = 0.318, respectively).

**Fig. 6:**
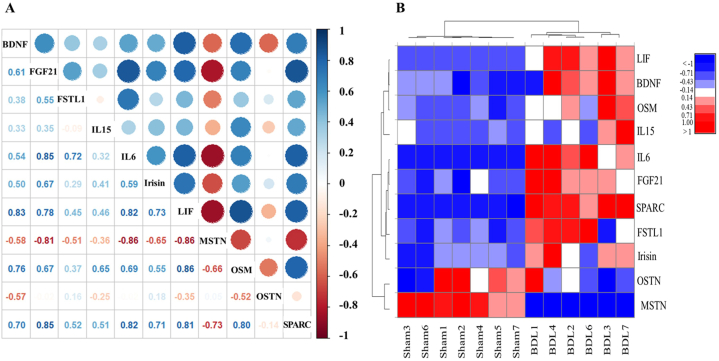
Correlation of adipo-myokines in sham control and BDL mice. (A) Spearman correlation analysis of 11 differentially expressed adipo-myokines. The red dots represent significant negative correlations, the blue dots indicate significant positive correlations, and the white color indicates no correlation. (B) Heatmap showing the level of adipo-myokines in each mouse. The increase in the relative level of adipo-myokines is represented by a transition from blue to white to red, as shown in the legend. BDL, bile duct ligation; BDNF, brain-derived neurotrophic factor; FGF21, fibroblast growth factor 21; FSTL1, follistatin-like protein 1; IL-15, interleukin-15; IL-6, interleukin-6; LIF, leukemia inhibitory factor; MSTN, myostatin; OSM, oncostatin M; OSTN, osteocrin/musclin; SPARC, secreted protein acidic and rich in cysteine. Sham: control mice that received sham surgery (n = 7); BDL: mice that received BDL surgery (n = 6).

In addition, we examined the correlation among adipo-myokines, including their levels in individual mice (Fig. 6A and B). Fig. 6A shows that MSTN and OSTN were the most strongly negatively correlated, whereas SPARC, LIF, and IL6 were all strongly positively correlated with each other. The heatmap demonstrated a strongly positive relationship between adipo-myokines in individual mice in the BDL group, e.g., BDL mouse #1 (OSTN, FGF21, IL6, and SPARC), BDL mouse #3 (SPARC, OSM, BDNF, and LIF), BDL mouse #4 (FGF21, IL6, irisin, and BDNF), BDL mouse #6 (FSTL1 and IL6), and BDL mouse #7 (SPARC and IL15) (Fig. 6B). Only SPARC showed an overall positive correlation in BDL mice. In sham control mice, only MSTN and OSTN showed positive correlations among the tested adipo-myokines; however, these two proteins showed negative correlations in BDL mice.

### 3.6 Correlation analysis between the gut microbiota and serum adipo-myokines

**Fig. 7:**
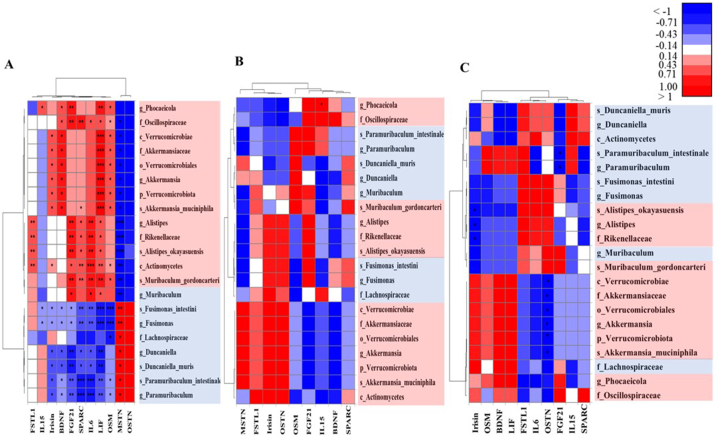
Heatmap analysis of the relationship of perturbed gut bacteria and altered adipo-myokines in all experimental mice (A), sham control mice (B), or BDL mice (C). The X-axis and Y-axis represent adipo-myokines (BDNF, irisin, FSTL1, FGF21, MSTN, OSTN, LIF, IL6, OSM, IL15, SPARC) and 21 taxa of the gut microbiota, respectively. The names with color indicate the taxa of the gut microbiota of sham control mice (blue) and BDL mice (red) with a LDA score ≥4.0. The color depth indicates the Spearman correlation, and the right-side legend shows the color interval of different values. BDL, bile duct ligation; BDNF, brain-derived neurotrophic factor; FGF21, fibroblast growth factor 21; FSTL1, follistatin-like protein 1; IL-15, interleukin-15; IL-6, interleukin-6; LIF, leukemia inhibitory factor; MSTN, myostatin; OSM, oncostatin M; OSTN, osteocrin/musclin; SPARC, secreted protein acidic and rich in cysteine. Sham: control mice that received sham surgery (n = 7); BDL: mice that received BDL surgery (n = 6). ∗*p* < 0.05, ∗∗*p* < 0.01, ∗∗∗*p* < 0.001; tested by Spearman's rank correlation.

To better understand the relationship between the fecal microbiota and serum adipo-myokines, Spearman's rank correlation analysis was performed on significant taxa detected in the gut microbiota and blood adipo-myokines (Fig. 7). In this analysis, 21 taxa with a LDA score >4.0 and *p* < 0.05 in Mann-Whitney *U* test were selected (Fig. 4D–Table 1). In all experimental mice, serum levels of MSTN and OSTN were positively correlated with the family *Lachnospiraceae*, the genera *Fusimonas*, *Duncaniella*, and *Paramuribaculum*, and the species *Duncaniella muris*, *Fusimonas intestini*, and *Paramuribaculum intestinale* in the gut (Fig. 7A). However, the high abundances of these taxa in the gut were negatively correlated with the serum levels of FGF21, IL6, SPARC, BDNF, irisin, LIF, and OSM. Moreover, significant positive correlations were identified between adipo-myokines such as FSTL1, FGF21, and IL6 and gut bacteria belonging to the class *Actinomycetes*, the family *Rikenellaceae*, the genus *Alistipes*, and the species *Alistipes okayasuensis.* High levels of FGF21 and SPARC were positively correlated with the class *Actinomycetes*, the family *Oscillospiraceae*, and the species *Muribaculum gordoncarteri* and negatively correlated with the genus *Paramuribaculum*, including the species *Paramuribaculum intestinale*. Notably, LIF showed a significant strong positive correlation with the phylum *Verrucomicrobiota*, which comprised only the species *Akkermansia muciniphila* (Fig. 7A).

For sham control mice, a significant positive correlation was observed between the *Phocaeicola* genus and IL15 (*p* = 0.048) (Fig. 7B). On the other hand, significant negative correlations between irisin and the family *Rikenellaceae*, the genus *Alistipes*, and the species *Alistipes okayasuensis* were observed in BDL mice. In addition, higher abundances of the phylum *Verrucomicrobiota*, the class *Verrucomicrobiae*, the order *Verrucomicrobiales*, the family *Akkermansiaceae*, the genus *Akkermansia*, and the species *Akkermansia muciniphila* were correlated with a lower serum level of OSTN, and a higher abundance of the genus *Paramuribaculum*, including the species *Paramuribaculum intestinale*, was negatively correlated with FGF21 (Fig. 7C) in sham control mice.

## 4 Discussion

Gut microbiota dysbiosis is a well-known phenomenon in experimental obstructive cholestasis; however, there is limited information on specific gut bacteria and their relevance to adipo-myokines in the blood in HE. In this study, we used a preclinical model of HE to investigate the association of circulating adipo-myokine levels with the microbiota composition of feces. We observed higher proportions of mucus-colonizing bacteria such as *A. muciniphila*, *A. okayasuensis*, and *M. gordoncarteri* in the gut of BDL mice, which showed no difference in alpha-diversity compared with that of sham control mice. Moreover, these three bacterial strains were significantly associated with certain adipo-myokines related to obesity or inflammation (LIF, FSTL1, FGF21, SPARC, and IL6) (Supplementary Fig. 4). Overall, the abundance of the phylum *Verrucomicrobiota,* including the species *A. muciniphila*, showed the strongest positive correlation with LIF in all experimental mice (*p* = 0.001), which also showed a positive tendency but no statistical significance in BDL mice (*p* = 0.103). In sham control mice, the *Phocaeicola* genus was positively correlated with IL15 (*p* = 0.048). The population of *A. muciniphila* was negatively correlated with OSTN. In addition, the genus *Paramuribaculum* including the species *Paramuribaculum intestinale* was negatively correlated with FGF21, and the genus *Alistipes*, the family *Rikenellaceae*, and the species *A. okayasuensis* were negatively correlated with irisin. These results may shed light on the effect of the gut microbiota on circulating adipo-myokine levels in liver complications caused by cholestatic liver injury.

Our results showed the marked elevation of ALT, AST, total and direct bilirubin, and ammonia levels in the plasma at 14 days after BDL surgery [28] implying the loss of integrity in the hepatocyte membrane, leading to the disruption of metabolism and leakage of liver enzymes. Mice with BDL surgery have been reported to show behavioral changes and liver function failure with hyperammonemia [29]. BDL-mediated changes in bile acid metabolism, systemic inflammation, and gut dysmotility may lead to intestinal dysbiosis [30]. Alaish et al. [31] found that changes in the intestinal microbiome in C57/B6J mice at late stages after BDL (14 days) were related to advanced fibrogenesis (cirrhosis). In this study, we found that the beta-diversity and fecal microbiota composition of BDL mice were significantly different from those of sham control mice. At the family level, we found that BDL surgery resulted in the enrichment of *Oscillospiraceae* (*Bacillota* [*Firmicutes*] phylum), *Akkermansiaceae* (*Verrucomicrobiota* phylum), *Bacteroidaceae* (*Bacteroidota* phylum), and *Rikenellaceae* (*Bacteroidota* phylum) in the mouse gut. The increased abundance of the *Rikenellaceae* family was previously observed in BDL mice; nevertheless, the change in the *Lachnospiraceae* family observed in our study was different from that previously reported by Yang et al. [32]. However, Korobeinikova et al. [33] reported that the abundance of *Oscillospiraceae* was enriched in control samples compared with non-alcoholic fatty liver disease (NAFLD) samples. Yan et al. [34] reported that the abundances of beneficial bacteria at the family level, such as *Rikenellaceae* and *Oscillospiraceae*, in the hepatitis B virus-associated liver cirrhosis (HBV-LC) and HBV-hepatic cellular carcinoma (HCC) groups were decreased.

We found that BDL surgery significantly increased the abundance of certain bacteria in the mouse gut. In the phylum *Verrucomicrobiota*, only the species *A. muciniphila* was significantly enriched in BDL mice. Previous studies demonstrated that *A. muciniphila* was more abundant in the microbiota of patients with Parkinson's disease or multiple sclerosis [35,36]. In addition, this bacterium was reported to be negatively correlated with body fat content, fat mass gain, and abdominal fat [37], which is consistent with our results showing that BDL mice appeared lean with severe body weight loss. On the other hand, a lower abundance of this bacterium was associated with multiple diseases including sarcopenia and liver inflammation in both mice and humans [38,39]. *A. muciniphila* has also been recognized as a promising next-generation beneficial strain due to its proven efficacy in improving intestinal barrier function [40] and metabolic diseases including obesity, hepatic steatosis, and intestinal inflammation in mice [37]. We found that *A. muciniphila* was positively correlated with LIF post-BDL surgery. LIF, involved in metabolic regulation, is known for its inhibition of food intake, causing weight loss and decreased serum leptin levels [41]. In a previous study, white adipose tissue and serum LIF levels were increased in obese mice and NAFLD patients, and circulating LIF levels showed a significant positive correlation with metabolic risk factors.

Furthermore, the relative abundance of the species of *M. gordoncarteri* was significantly enriched in BDL mice. *M. gordoncarteri*, an anaerobic bacterial strain, was recently isolated from the fecal pellets of conventionally raised C57BL/6J mice [42] and has not been associated with a disease. Previous studies on the *Muribaculaceae* family showed that an increase in the relative abundance of *Muribaculaceae* was associated with acute pancreatitis in mice [43] and NAFLD in humans [33]. In a colitis mouse model induced by dextran sulfate sodium, the relative abundance of *Muribaculaceae* was negatively correlated with pro-inflammatory cytokines and positively correlated with the expression levels of tight junction proteins and mucin 2 [44]. Therefore, bacterial species belonging to *Muribaculum* may be important for maintaining normal conditions in the mouse gut. We also found that *M. gordoncarteri* was positively correlated with FGF21 in all experimental mice. FGF21 is expressed predominantly in the liver [45] but also in the adipose tissue, muscle, and pancreas [[46], [47], [48], [49]]. More importantly, several recent studies shed light on the role of FGF21 as an endogenous regulator of tissue homeostasis by keeping tissue inflammation of both metabolic and non-metabolic origins and subsequent tissue deterioration and damage at bay [50]. FGF21 is a promising intervention therapy for metabolic diseases such as fatty liver, obesity, and diabetes, which is an inducible hepatokine that may be used as a biomarker for normal hepatocyte function.

*A. okayasuensis* was also significantly enriched in BDL mice. Förster et al. [51] reported that the previously undescribed mouse gut bacteria *Duncaniella muricolitica* and *A. okayasuensis can* affect outcomes in a DSS model of colitis and may act as pathobionts. According to the Taxonomy Database of The National Center for Biotechnology Information (NCBI; txid239759), as of January 2024, the genus *Alistipes* consists of 18 species and has been implicated in liver fibrosis, colorectal cancer, cardiovascular disease, HE recurrence, and mood disorders [52]. In this study, *A. okayasuensis* showed a positive correlation with FSTL1. FSTL1 expression is enhanced in the liver tissue and serum of patients with advanced fibrosis and steatosis [53]. Although studies on the role of FSTL1 in inflammation have been widely conducted in the past decade [54], whether FSTL1 plays a proinflammatory role is still unclear [[54], [55], [56], [57], [58]]. In this study, the serum FSTL1 level was significantly higher in BDL mice than in sham control mice, which is in agreement with the findings of a previous study [59] reporting high levels of FSTL1 expression in the macrophages of human or mouse liver fibrotic tissues.

The relative abundances of *A. okayasuensis*, *A. muciniphila*, and *M. gordoncarteri* (statistically significant in Mann-Whitney *U* test and LEfSe analysis) were examined in mice with liver injury induced by BDL. Interestingly, these three BDL-induced gut bacteria may affect the mucus layer. *A. muciniphila* is a mucin-degrading bacterium, which can stimulate mucin production, increase intestinal mucous layer thickness and intestinal barrier integrity [37], and protect the intestinal tract from pathogens through competitive rejection [60]. *Muribaculaceae* (family of *A. okayasuensis*) has been shown to be a major utilizer of mucus-derived monosaccharides in the gut [61]. Grade 1–2 mucositis has been associated with the enrichment of *Eubacterium rectale*, *Alistipes putredinis*, and *Ruminococcaceae* family members [62].

Adipo-myokine studies with a BDL mouse model can help identify specific disease-related factors. We demonstrated that some adipo-myokines, such as BDNF, FGF21, FSTL1, IL6, irisin, LIF, OSM, and SPARC, were significantly higher in BDL mice than in sham control mice. BDNF is known to play an important role in the survival, maintenance, and growth of neurons [63], and it is important for regulating muscle regeneration following muscle injury [64]. Our results revealed that a higher level of serum BDNF was detected in BDL mice with body weight loss compared with sham control mice. The results are in agreement with the findings of previous research conducted on adults, which showed the inverse relationship of BDNF with body weight [65]. IL6, OSM, and LIF belong to the IL6 family. IL-6 levels are very low under normal conditions but can increase up to several thousand-fold in inflammatory states [66]. Previous studies reported that an increase in serum IL-6 levels in BDL rats without any obvious signs of infection may suggest the activation of the pro-inflammatory response similar to that observed in cirrhotic patients [67,68]. OSM plays several unique roles in anti-inflammation, metabolic function, regeneration, and hematopoiesis [69], which are not shared with the other IL-6 family cytokines. Although the mechanisms underlying OSM-dependent fibrogenesis remain unclear, it was reported that OSM expression is elevated in both the liver and serum of patients with LC or HCC [70,71]. In addition, Yuan et al. detected higher levels of serum LIF in patients with simple steatosis and NASH, as well as in high-fat-fed and *ob/ob* mice [72]. Our results are in agreement with previous findings showing an increase in serum IL-6, OSM, and LIF levels in BDL rats without any obvious signs of infection. Irisin, a cleaved form of fibronectin type III domain-containing protein 5 (FNDC5) [73], was first reported as a potential mediator of the beneficial effect of exercise [74]. Armandi et al. [75] found that irisin levels were significantly higher in individuals with significant fibrosis. In this study, we detected higher serum irisin levels in the BDL group with body weight loss. However, Kukla et al. reported that serum irisin levels were not significantly different between LC patients with and without type 2 diabetes and were not correlated with sarcopenia [76]. SPARC, also known as osteonectin or BM-40, is an extracellular matrix-associated protein ubiquitously expressed in most tissues, especially subcutaneous fat [77]. SPARC is known to inhibit adipogenesis [78] and promote adipose tissue fibrosis. In addition, SPARC is involved in various biological processes including liver fibrogenesis; however, its role in acute liver damage is unknown. We found that mean serum SPARC levels were significantly higher in BDL mice than in sham control mice. This result is in agreement with previous findings showing that SPARC was overexpressed in cirrhotic livers from mice and patients [79]. MSTN is a negative regulator of muscle mass, which also negatively modulates the activity of the Akt pathway [80]. Giusto et al. [81] reported that an increase in MSTN expression was associated with a decrease in AKT-mTOR expression in BDL mice, and skeletal muscle myopenia was present in experimental models of BDL-induced cirrhosis. However, Alexopoulos et al. [82] reported that MSTN values were low in cirrhotic patients with sarcopenia and were decreased further in severe sarcopenia. In our study, MSTN was not detected in the serum of all BDL mice. Further investigation is needed to determine if this outcome is caused by the very low secretion levels of MSTN or sensitivity of the experimental kit. Although the metabolic mechanism of adipo-myokines in BDL-induced liver injury has not been elucidated, we believe that adipo-myokine expression may be associated with cholestatic liver injury, which can develop into HE.

In this study, we determined the correlation between the gut microbiota in BDL mice and serum adipo-myokines. We identified 8 taxa (the class *Actinomycetes*, the families *Oscillospiraceae and Rikenellaceae*, the genera *Alistipes* and *Phocaeicola*, and the species *Alistipes okayasuensis*, *Muribaculum gordoncarteri*, and *Akkermansia muciniphila*) showing significant positive correlations with more than 3 factors among the assessed adipo-myokines. In addition, we observed that FSTL1, FGF21, and IL6, which may be related to obesity and inflammation [83], were positively correlated with each other and with the class *Actinomycetes*, the family *Rikenellaceae*, the genus *Alistipes*, and the species *A. okayasuensis.* The class *Actinomycetes* ranges from harmless bacteria to pathogens that are extremely useful for antibiotic producers. In this study, *Actinomycetes* showed positive correlations with most of the tested adipo-myokines, which mainly consisted of *Bifidobacterium pseudolongum* and *Corynebacterium casei*. In addition, the abundance of the family *Rikenellaceae* in our study was attributed to *A. okayasuensis,* a recently isolated and identified pathogen in a DSS model of colitis [51]. Further studies on *A. okayasuensis* in an obstructive cholestatic injury model are needed. SPARC has recently attracted substantial interest due to its roles in obesity, insulin resistance, and metabolic syndrome [77], and FGF21 is thought to have therapeutic potential for obesity, T2DM, and NAFLD [84]. Based on our results, the class *Actinomycetes,* the family *Oscillospiraceae,* and the species *M. gordoncarteri* showed positive correlations with SPARC and FGF21. Interestingly, *A. muciniphila* demonstrated a strong positive correlation with LIF. LIF is an important regulator of liver TG homeostasis [72] and plays important roles in the regulation of different physiological and pathological processes. LIF induces the activation of signal transducer and activator of transcription (STAT) and mitogen-activated protein kinase (MAPK) pathways by binding to its receptor (LIF receptor) [72]. In addition, recent studies suggested that LIF might be involved in metabolic regulation, which was identified as a tumor-secreted molecule that could promote adipose tissue and body weight loss in cachectic settings [72]. In this study, we found that the abundance of the genus *Paramuribaculum*, including the species *Paramuribaculum intestinale*, was negatively correlated with FGF21 in BDL mice*,* which is regarded as a promising intervention therapy for obesity [84].

Systemic inflammation has been established as a major contributing factor to HE pathogenesis, synergizing with ammonia to enhance neurotoxicity via increased blood-brain-barrier permeability and cerebral oxidative stress [85].

This study has several limitations. First, this study focused on correlations between the gut microbiota and adipokine levels but could not explain the causality. In the future, further experiments are required to manipulate the gut microbiota, such as antibiotic treatment or fecal microbiota transplantation, to investigate the causal role of specific taxa in adipokine level changes. Second, mechanistic studies are needed to elucidate the pathways by which gut microbiota changes influence adipokine production, for example, by investigating the effects of specific bacterial metabolites on liver cells. Third, we conducted the experiments using 6–7 mice in each group based on previous studies [[86], [87], [88], [89]]; however, the number of mice should be increased to clarify the results.

## 5 Conclusions

Despite the study limitations, our results suggest that *A. muciniphila*, *A. okayasuensis*, and *M. gordoncarteri* may play key roles in HE or sarcopenia with cholestasis. Further studies with various metabolic parameters are needed to determine whether the three strains (*A. muciniphila*, *Alistipes okayasuensis*, and *Muribaculum gordoncarteri*) can act as biomarkers in BDL-induced cholestatic liver disease-related HE.

## Availability of data and materials

The sequencing data that support the findings of this study are openly available in the NCBI BioProject database with the project identification number PRJNA983098 (sample accession numbers: SAMN36269388 to SAMN36269399).

## Funding

This study was supported by the 10.13039/501100003725National Research Foundation of Korea (NRF grant 2022R1A2C1006125 to Juhyun Song and 2022R1A2C1010398 to Oh Yoen Kim).

## CRediT authorship contribution statement

**Bokyung Lee:** Writing – original draft, Visualization, Validation, Software, Methodology, Investigation, Formal analysis, Data curation. **Danbi Jo:** Visualization, Validation, Methodology, Investigation, Formal analysis, Data curation. **Jihyun Park:** Methodology, Investigation. **Oh Yoen Kim:** Writing – review & editing, Writing – original draft, Visualization, Validation, Supervision, Methodology, Investigation, Funding acquisition, Data curation, Conceptualization. **Juhyun Song:** Writing – review & editing, Writing – original draft, Visualization, Validation, Supervision, Software, Resources, Methodology, Investigation, Funding acquisition, Formal analysis, Data curation, Conceptualization.

## Declaration of competing interest

No potential conflict of interest was reported by the author(s)
