# Supplementary material for: Gut microbiota and their relationship with circulating adipokines in an acute hepatic encephalopathy mouse model induced by surgical bile duct ligation
Source: Heliyon. 2024 Sep 26;10(19):e38534. doi: 10.1016/j.heliyon.2024.e38534 (PMC11466606; doi:10.1016/j.heliyon.2024.e38534)
Supplement: Multimedia component 1 [file mmc1.docx]

**Supplementary Figures**

**
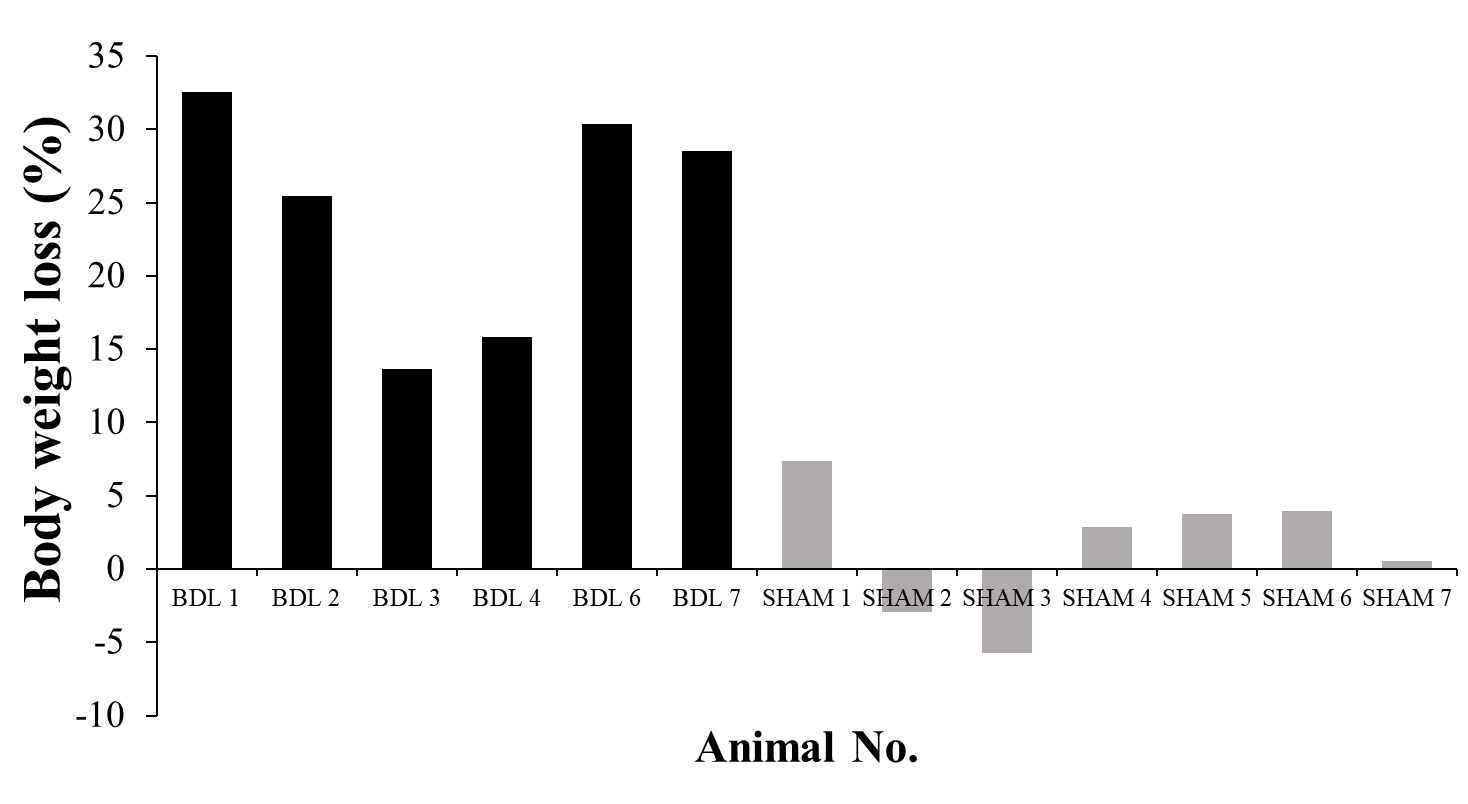
**

**Supplement Figure 1.** **Body weight change (%) between sham controls and BDL mice before after the surgery**

Surgery was conducted on day 0, and mice were sacrificed on day 14. Body weight loss (%) was calculated as [(body weight at 0 day - body weights at 14 day)/ body weight at 0day]x100. Sham: sham control which received sham surgery (n = 7); BDL: mice which received BDL surgery (n = 6)


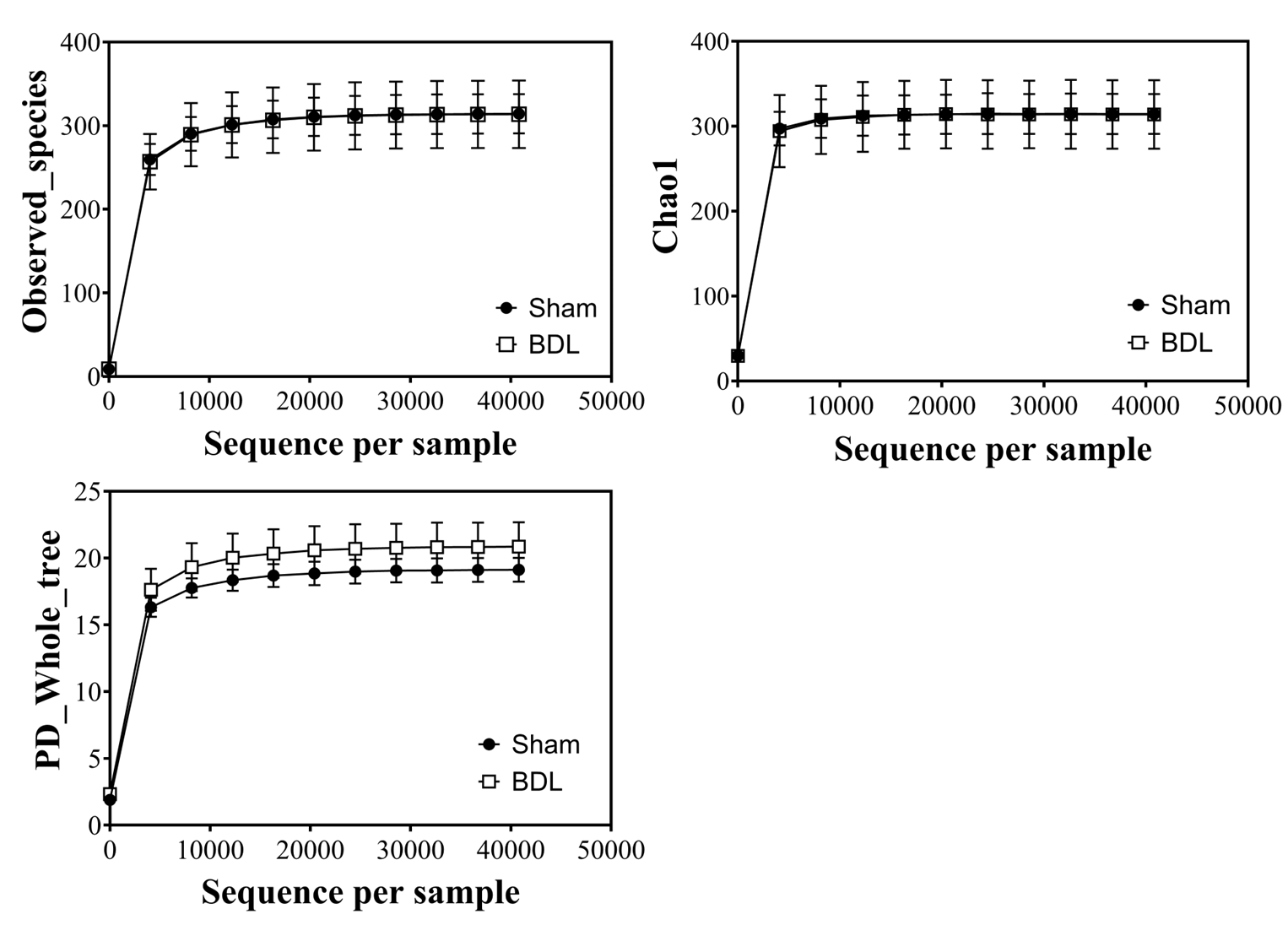


**Supplement Figure 2. Alpha rarefaction curves based on observed number.**

Rarefaction curves based on observed species value(A), Chao1(B)and PD whole tree(C). Data are presented as means ± SEM. The rarefaction curve was plotted where the X-axis represents the number of clones (sequences) and the Y-axis represents the number of ASVs. Sham: sham control which received sham surgery (n = 7); BDL: mice which received BDL surgery (n = 6).


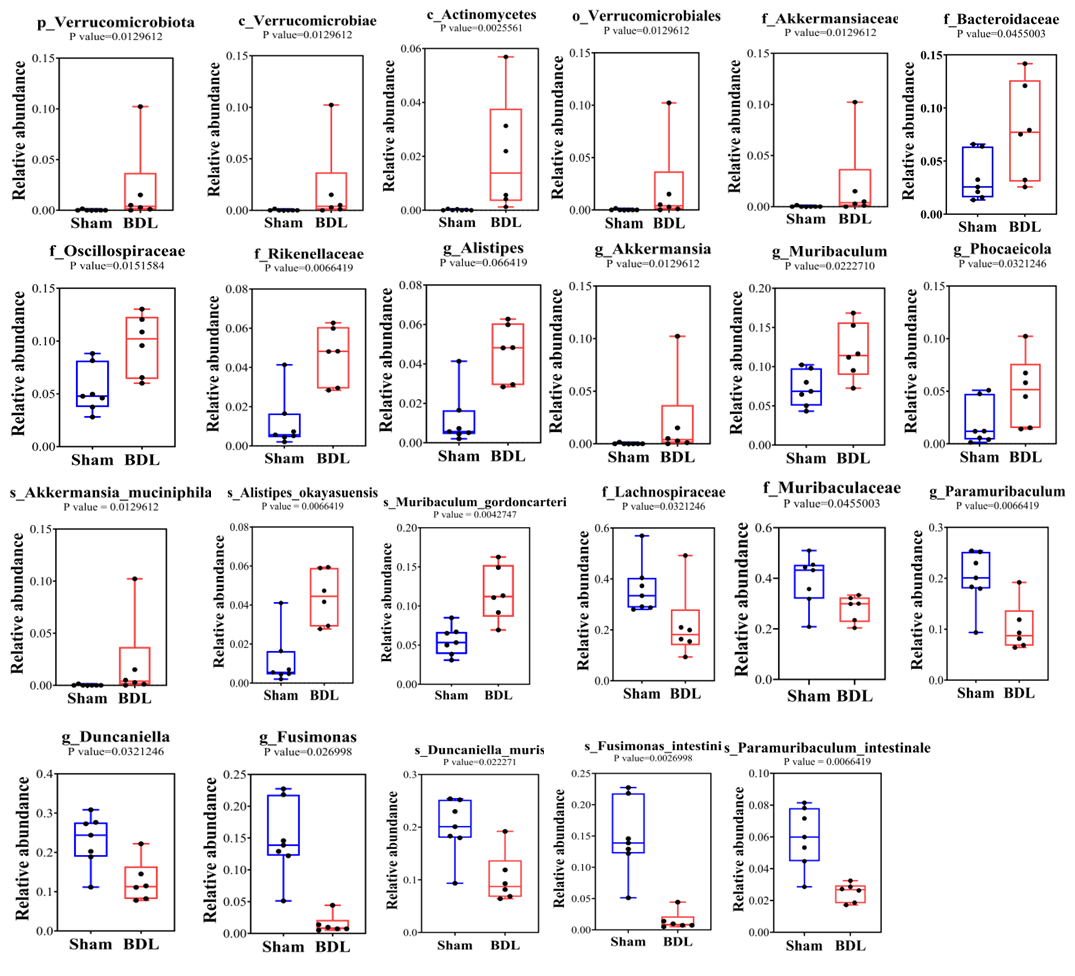


**Supplementary Figure 3. Relative abundance of gut microbiota compared with LDA score ≥ 4.**

Sequences were analyzed using the QIIME2 (Quantitative Insights into Microbial Ecology). Each box plot represents the median, interquartile range, minimum, and maximum values. Tested by Kruskal Wallis test. Sham (blue box): sham control which received sham surgery (n = 7); BDL (red box): mice which received BDL surgery (n = 6).


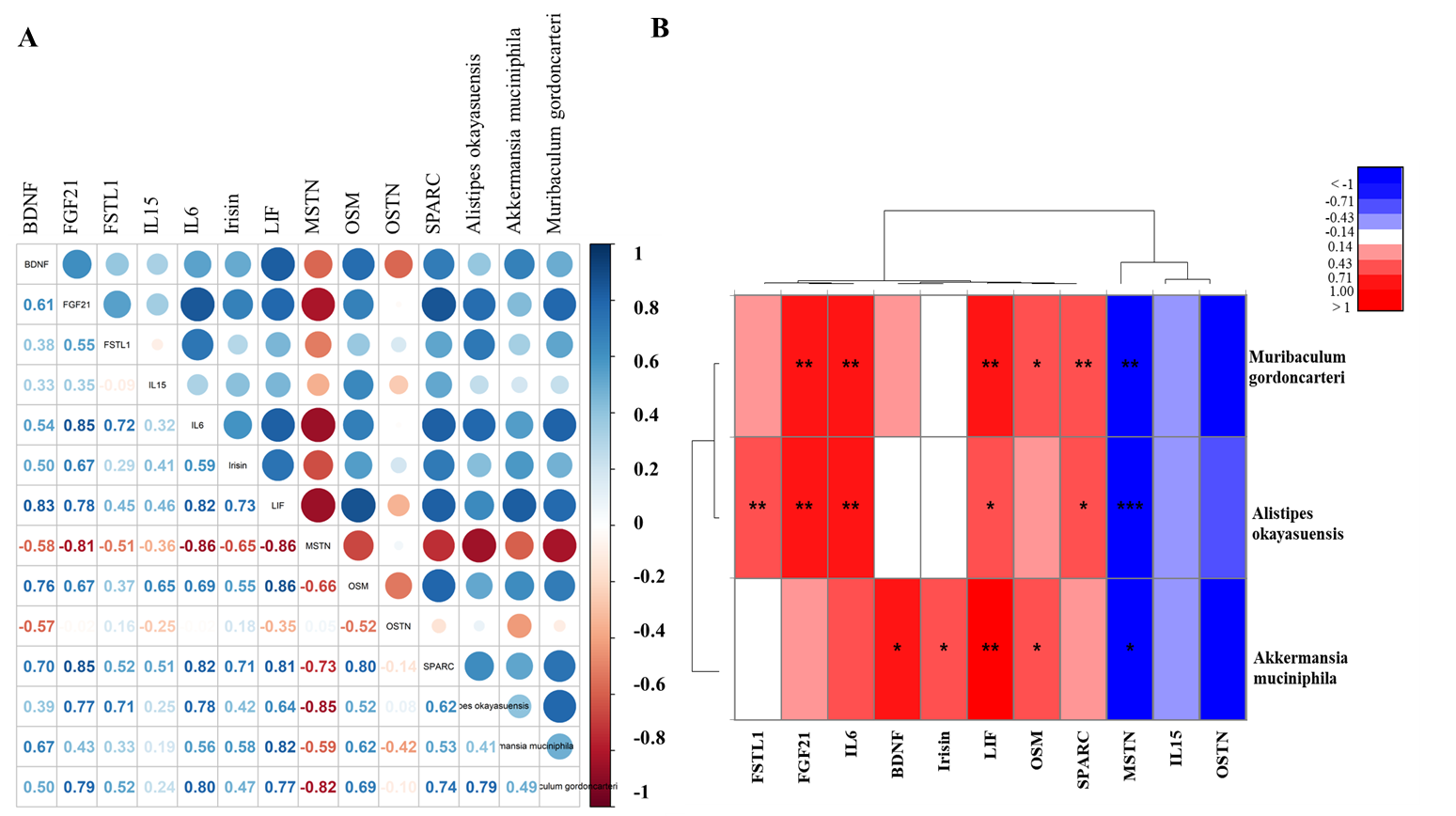


**Supplementary Figure 4. Correlation of adipomyokine and 3 potential BDL-related gut microbiota biomarkers.
(**A) Spearman correlation analysis of the 11 differentially expressed adipo-myokines and 3 potential BDL-related gut microbiota biomarkers. The red dots represent the significantly negative correlation, the blue dots indicate the significantly positive correlation, and white color indicates no correlation. (B) Heatmap showing spearman’s correlation matrix of adipo-myokines and 3 potential BDL-related gut microbiota biomarkers. The increase in the relative level of adipo-myokines is represented by a transition from blue to white to red, as shown in the legend. *p < 0.05, **p < 0.01 and ***p < 0.001; tested by student t-test. BDL, bile duct ligation; BDNF, brain-derived neurotrophic factor; FGF21, fibroblast growth factor 21; FSTL1, follistatin-like protein 1; IL-15, interleukin-15; IL-6, interleukin-6; LIF, leukemia inhibitory factor; MSTN, myostatin; OSM, oncostatin M; OSTN, osteocrin/musclin; SPARC, secreted protein acidic and rich in cysteine. Sham: sham control which received sham surgery (n = 7); BDL: mice which received BDL surgery (n = 6).
